# Six underlying health conditions strongly influence mortality based on pneumonia severity in an ageing population of Japan: a prospective cohort study

**DOI:** 10.1186/s12890-018-0648-y

**Published:** 2018-05-23

**Authors:** Sugihiro Hamaguchi, Motoi Suzuki, Kota Sasaki, Masahiko Abe, Takao Wakabayashi, Eiichiro Sando, Makito Yaegashi, Shimpei Morimoto, Norichika Asoh, Naohisa Hamashige, Masahiro Aoshima, Koya Ariyoshi, Konosuke Morimoto, on behalf of The Adult Pneumonia Study Group – Japan, Bhim Gopal Dhoubhadel, Akitsugu Furumoto, Masayuki Ishida, Tomoko Ishifuji, Satoshi Kakiuchi, Shungo Katoh, Emi Kitashoji, Takaharu Shimazaki, Masahiro Takaki, Kiwao Watanabe, Lay-Myint Yoshida, Hiroki Nanba, Naoto Hosokawa, Norihiro Kaneko, Hidenori Katsura, Naoko Katsurada, Kei Nakashima, Yoshihito Otsuka, Daisuke Suzuki, Kenzo Tanaka, Masayuki Chikamori, Hiroshi Nakaoka, Hiroyuki Ito, Kei Matsuki, Yoshiko Tsuchihashi

**Affiliations:** 1 Department of General Internal Medicine, Fukushima Medical University, Fukushima, Japan; 2 Department of Clinical Medicine, Institute of Tropical Medicine, Nagasaki University, Nagasaki, Japan; 3 Department of Laboratory Medicine, Ebetsu City Hospital, Ebetsu, Japan; 4 Department of General Internal Medicine, Ebetsu City Hospital, Ebetsu, Japan; 5 Department of General Medicine, Sapporo Hokushin Hospital, Sapporo, Japan; 6 Department of General Internal Medicine, Kameda Medical Centre, Kamogawa, Japan; 7 Department of Pulmonology, Kameda Medical Centre, Kamogawa, Japan; 8 Innovation platform & office for precision medicine, Nagasaki University Graduate School of Biomedical Sciences, Nagasaki, Japan; 9 Department of Internal Medicine, Juzenkai Hospital, Nagasaki, Japan; 10 Department of Internal Medicine, Chikamori Hospital, Kochi, Japan

**Keywords:** Underlying health conditions, Adult pneumonia, Ageing population, Mortality prediction

## Abstract

**Background:**

Mortality prediction of pneumonia by severity scores in patients with multiple underlying health conditions has not fully been investigated. This prospective cohort study is to identify mortality-associated underlying health conditions and to analyse their influence on severity-based pneumonia mortality prediction.

**Methods:**

Adult patients with community-acquired pneumonia or healthcare-associated pneumonia (HCAP) who visited four community hospitals between September 2011 and January 2013 were enrolled. Candidate underlying health conditions, including demographic and clinical characteristics, were incorporated into the logistic regression models, along with CURB (confusion, elevated urea nitrogen, tachypnoea, and hypotension) score as a measure of disease severity. The areas under the receiver operating characteristic curves (AUROC) of the predictive index based on significant underlying health conditions was compared to that of CURB65 (CURB and age ≥ 65) score or Pneumonia severity index (PSI). Mortality association between disease severity and the number of underlying health conditions was analysed.

**Results:**

In total 1772 patients were eligible for analysis, of which 140 (7.9%) died within 30 days. Six underlying health conditions were independently associated: home care (adjusted odds ratio, 5.84; 95% confidence interval, CI, 2.28–14.99), recent hospitalization (2.21; 1.36–3.60), age ≥ 85 years (2.15; 1.08–4.28), low body mass index (1.99, 1.25–3.16), neoplastic disease (1.82; 1.17–2.85), and male gender (1.78; 1.16–2.75). The predictive index based on these conditions alone had a significantly or marginally higher AUROC than that based on CURB65 score (0.78 vs 0.66, *p* = 0.02) or PSI (0.78 vs 0.71, *p* = 0.05), respectively. Compared to this index, the AUROC of the total score consisting of six underlying health conditions and CURB score (range 0–10) did not improve mortality predictions (*p* = 0.3). In patients with one or less underlying health conditions, the mortality was discretely associated with severe pneumonia (CURB65 ≥ 3) (risk ratio: 7.24, 95%CI: 3.08–25.13), whereas in patients with 2 or more underlying health conditions, the mortality association with severe pneumonia was not detected (risk ratio: 1.53, 95% CI: 0.94–2.50).

**Conclusions:**

Mortality prediction based on pneumonia severity scores is highly influenced by the accumulating number of underlying health conditions in an ageing society. The validation using a different cohort is necessary to generalise the conclusion.

**Electronic supplementary material:**

The online version of this article (10.1186/s12890-018-0648-y) contains supplementary material, which is available to authorized users.

## Background

The burden of pneumonia is increasing in societies with ageing populations, despite guideline-based standard management [1, 2]. In Japan, people have direct and around-the-clock access to high quality medical care under the Universal National Health Insurance Coverage, but pneumonia mortality is steadily rising and the disease is now ranked third as a cause of death [3]. In a society with an ageing population, pneumonia death rates show two major distributions, with one subgroup containing previously healthy patients with overwhelming septic shock or multiple organ failure, and the other containing patients with multiple underlying health conditions for death [4]. This rise in mortality is thought to be due to the increase in the latter subgroup [5, 6]. Most of underlying health conditions are age-related chronic factors, such as comorbid illnesses, swallowing dysfunction, healthcare-associated morbidities or changes in immune function [7–10], which are not easily or quickly modified by treatment.

An accumulating number of studies have evaluated severity scores, ie. CURB65 (confusion, elevated blood urea nitrogen, tachypnoea, hypotension, and age ≥ 65) or Pneumonia Severity Index (PSI), for mortality prediction and severity-stratified decision-making for hospitalisation [11–14]. Predictor variables used in those models have mainly been parameters directly related to pneumonia severity, such as respiratory rate, blood pressure, consciousness level, oxygen saturation, or several laboratory or radiological test results, all of which are modifiable by appropriate management of pneumonia. However, in a population setting where a significant number of people have multiple underlying health conditions, we hypothesised that pneumonia severity scores alone should have a limitation of predicting mortality, and that co-evaluating underlying health conditions which patients already have before they contract pneumonia should provide more comprehensive mortality assessment. We found no study exclusively evaluating the influence of underlying health conditions on severity-based pneumonia mortality prediction.

This study is aimed to identify mortality-associated underlying health conditions independent of pneumonia severity among adult pneumonia patients and to evaluate how these conditions influence on mortality prediction based on pneumonia severity scores. We analysed data from a cohort of patients enrolled in a prospective multicentre surveillance for community-acquired pneumonia (CAP) and healthcare-associated pneumonia (HCAP) in Japan [15]. We focused on clinical conditions obtainable by simple history-taking and basic examination at initial patient contact, making the results applicable in primary clinical settings, including busy emergency rooms.

## Methods

### Study setting, design, and sites

The Adult Pneumonia Study of Japan was a two-year prospective multicentre study which began in September 2011 at four community hospitals in Japan. [15]

According to the national statistics in 2012, 25.1 and 3.6% of the Japanese population were aged ≥65 and ≥ 85 years, respectively [3]. The estimated coverage rate of the 23-valent polysaccharide pneumococcal vaccine for adults was 17.5% in 2012 [16]. Initial empiric antibiotic treatment for CAP and HCAP is informed by the guidelines of The Japanese Respiratory Society, which generally follow the international guidelines [17]. The current analysis was based on a dataset which had been used in our previous work [15]: the data had been collected between September 2011 and January 2013.

### Patient enrollment

All patients who visited the outpatient department of or were admitted to our hospitals were enrolled if they fulfilled all of the following criteria: 1) aged ≥15; 2) symptomology compatible with pneumonia (e.g., fever, cough, sputum, pleuritic chest pain, dyspnoea); 3) new pulmonary infiltrates by chest X-ray (CXR) or computed tomography (CT) scan images consistent with pneumonia. All CXR and CT scan images were reviewed by multiple clinicians on-site and consensus interpretations were recorded. If a patient developed the disease more than 48 h after hospitalisation, the patient was classified as having hospital-acquired pneumonia [18] and was not enrolled. Repeated episodes of pneumonia in the same patient within a two-week period after enrolment were regarded as a single episode.

### Data collection

Demographic and clinical data were collected through direct interviews of patients or their guardians and from reviews of medical charts and laboratory databases. Data on patient background, comorbid illnesses, risk factors for aspiration-associated pneumonia, symptoms, physical signs, laboratory and radiological results, therapeutic information, and outcomes were collected.

### Definitions

HCAP was defined, based on the American Thoracic Society and Infectious Disease Society of America criteria, as pneumonia in any patient who met at least one of the following criteria: hospitalisation for ≥2 days in the preceding 90 days; residence in a nursing home or extended care facility; home infusion therapy; chronic dialysis within 30 days; and home wound care [19]. We did not use the criterion of family member infection with a multidrug resistant pathogen because of the difficulty in obtaining this information through history-taking in the study setting. Home infusion therapy and home wound care were combined into a “home care” variable. Cases of pneumonia that did not meet the HCAP criteria were defined as CAP.

### Underlying health conditions

We defined underlying health conditions as ageing-related or chronic conditions which patients had already had before they contracted pneumonia. Especially for elderly people, those conditions are practically difficult to remove or modify. Candidate conditions wer**e** selected a priori from those previously reported as mortality-associated factors [5, 8, 10, 20–22]: age; sex; HCAP conditions (hospitalization ≥2 days in the preceding 90 days, nursing home residency, home infusion therapy, chronic dialysis within 30 days, and home wound care); comorbid illnesses (congestive heart failure, liver disease, renal disease, neoplastic disease, chronic lung diseases, diabetes mellitus, and dementia); risk factors for aspiration pneumonia (witnessed aspiration, chronically impaired conscious level, chronic neurologic disorders, tube feeding, and bed-ridden state); and body mass index (BMI) (low: < 18.5, normal: 18.5–24.9, and high: ≥25). Comorbid illnesses were defined as chronic active medical conditions for which the patient received treatment or regular follow-up at the time of the enrolment.

### Severity assessment of pneumonia

CURB65 ≥ 3 was defined as severe pneumonia. For the exploratory analysis to identify independent mortality-associated factors, we used CURB score after removing the age index (≥65 years old) from the CURB65 scoring system [12] because we considered the “age” factor not as a disease parameter but as an underlying health condition. The CURB score is known as the modified British Thoracic Society scoring model [23]. It consists of a number obtained by the summation of four findings: confusion, blood urea nitrogen (BUN) ≥7 mmol/L, respiratory rate > 30 per minute, and blood pressure (< 90 mmHg in systole or ≤ 60 mmHg in diastole), all of which are pneumonia-related parameters and can be obtained by simple history-taking and basic examination. CURB score ≥ 2 was defined as severe pneumonia [23]. For the exploratory analysis, we did not use PSI scoring system, another widely used model, as a disease severity measure in our model because it consists of as many as 19 parameters, some of which, arterial blood gas analysis for instance, were not routinely measured in primary care settings [11].

### Statistical analysis

We categorised age into four ordinal age groups (≤64, 65–74, 75–84, and ≥ 85 years old). Categorical variables were summarised as frequencies and percentages. Pearson’s χ2 test was used for the analysis of discrete variables and comparisons of areas under the receiver operating characteristic curves (AUROC). Logistic regression models were used for both univariate and multivariate analyses to identify significant risk factors for 30-day mortality, and effect sizes were shown as odds ratios (OR) with a 95% confidence interval (95% CI). In the multivariate analysis, candidate underlying health conditions and CURB score were incorporated into the primary model. The number of missing values was not negligible in certain variables such as the CURB65 score (23.2%) and BMI (24.9%). We coded those missing values as “unknown status” and included all patients in our analysis. Explanatory variables with *p*-values less than 0.2 were first retained in the model by a backward stepwise method and incorporated into the final logistic regression model to identify independent mortality-associated factors and only variables with p-value < 0.05 were selected. To complement the lack of an external validation dataset, we assessed an internal validation with bootstrapping as a sensitivity analysis.

An AUROC for the mortality prediction index using the significantly associated underlying health conditions was calculated to evaluate the performance of the models for mortality prediction. Because CURB score is calculated by a simple summation of the number of four parameters without weighting according to the effect size of each significant variable, we also used a simple summation of the number of significant underlying health conditions as a predictive index for mortality. The performance of the underlying health condition index was evaluated in comparison to widely used severity indices, CURB65 and PSI, by calculating the AUROC of each [24]. To compare the performance, we used cases without missing variables so that CURB65 and PSI of all cases could be calculated.

To evaluate the influence of significant underlying health conditions depending on the severity of pneumonia, we first developed a prediction model exclusively consisting of underlying health conditions. Then, we dichotomised significant underlying health conditions according to the number of them into none or single underlying health condition or ≥ 2 underlying health conditions (multiple conditions), and compared the mortality of the two categories according to the severity of pneumonia.

All tests were two-tailed and a *p*-value less than 0.05 was regarded as statistically significant. STATA Version 13 (StataCorp LP) was used for statistical analysis.

### Ethical consideration

The study was conducted in accordance with the Guideline for Ethical Aspects in Epidemiological Study (Ministry of Health, Labour and Welfare, Japan 2008). The study was approved by the Institutional Review Board (IRB) of the Institute of Tropical Medicine at Nagasaki University and the IRBs of four participating hospitals: Ebetsu City Hospital, Kameda Medical Centre, Chikamori Hospital, and Juzenkai Hospital. Our hospital doctors and nurses verbally informed eligible patients and their guardians of the study objectives during their consultations. We also provided patients and their guardians with necessary information using a standardised questionnaire form. Informed consent to participate in the study was obtained from all participants. Written informed consent was obtained from the majority of the participants or their guardians and verbal consent was obtained from the rest of the participants. The requirement for obtaining written consent from all participants was waived by all IRBs because of the study’s observational nature without any deviation from current medical practice in accordance with the Guideline for Ethical Aspects in Epidemiological Study of Japan. Anonymised data were used for the analysis.

## Results

### Baseline characteristics and mortality

**Fig. 1:**
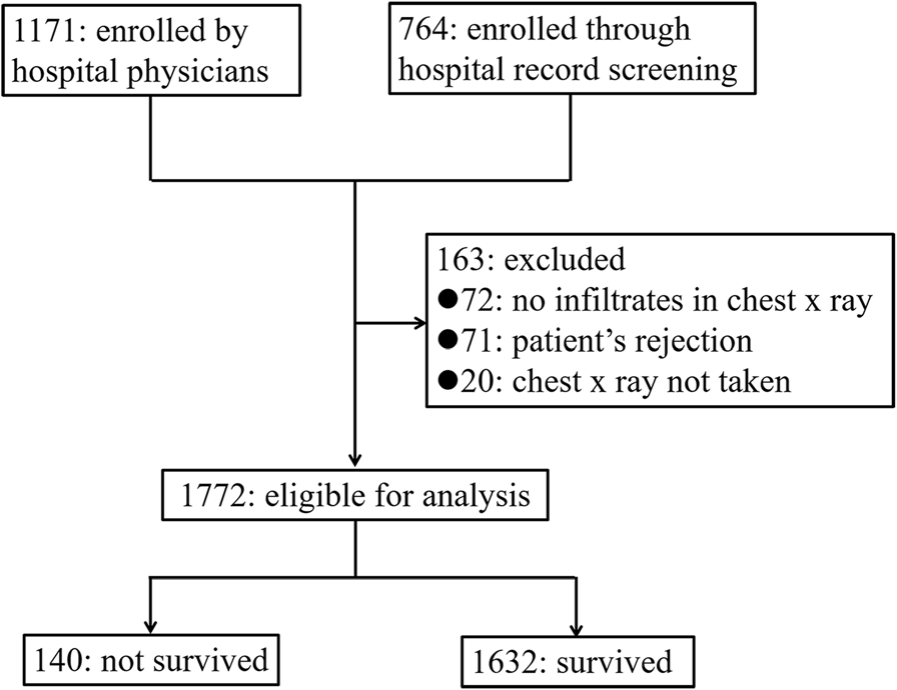
Enrolment and investigation flow. Enrolment and investigation flow after patients with no chest X ray (CXR) taken and no infiltrate on CXR were excluded, 1772 patients were eligible for the analysis, and 140 patients (7.9%) died within 30 days

A total of 1935 patients were enrolled. After 163 patients who did not meet the criteria (patient’s rejection, *n* = 71; CXR not taken, *n* = 20; no infiltrate on CXR, *n* = 72) were excluded, 1772 patients were eligible for the current analysis, and 140 patients (7.9%) died within 30 days of their first visit (Fig. 1).

Baseline characteristics of eligible patients are summarised in Table 1. The majority of the patients (*n* = 1328; 74.9%) were aged ≥65 years, with a median age of 77 (interquartile range: 64, 85) years old. Four hundred fifty patients (25.4%) were aged ≥85 years, and 1040 (58.7%) were male. Five hundred eighty-five patients (33.0%) were classified as HCAP, mostly due to recent hospitalisation and/or nursing home residency. The most common comorbid illnesses were chronic lung disease (22.9%), followed by diabetes mellitus (19.2%) and neoplastic disease (18.5%). Overall, 39.3% of patients had at least one risk factor for aspiration pneumonia, such as chronic neurologic disorders; 21.6% were underweight (BMI < 18.5); and 27.0% had a CURB score of two or more.

**Table 1:** Baseline characteristics of the survivors and non-survivors

	Number of survivors	Number of non-survivors^a^
	*N* = 1632 (92.1%)	*N* = 140 (7.9%)
	n (%)	n (%)
Underlying health conditions		
Age group		
≤ 64	429 (96.6)	15 (3.4)
65–74	291 (92.1)	25 (7.9)
75–84	517 (92.0)	45 (8.0)
≥ 85	395 (87.8)	55 (12.2)
HCAP factors		
Hospitalisation ≥2 days in the preceding 90 days, *N* = 1770	238 (83.5)	47 (16.5)
Nursing home resident, *N* = 1771	225 (86.2)	36 (13.8)
Chronic dialysis within 30 days, *N* = 1547	24 (82.8)	5 (17.2)
Home care, *N* = 1552	14 (66.7)	7 (33.3)
Home infusion therapy	5 (62.5)	3 (37.5)
Home wound care	9 (69.2)	4 (30.8)
Male gender	940 (90.4)	100 (9.6)
Comobidities		
Congestive heart failure	212 (89.1)	26 (10.9)
Liver disease	83 (87.4)	12 (12.6)
Renal disease	142 (88.7)	18 (11.3)
Neoplastic disease	284 (86.6)	44 (13.4)
Chronic lung disease	368 (90.6)	38 (9.4)
Diabetes mellitus	309 (90.9)	31 (9.1)
Dementia	202 (88.2)	27 (11.8)
Risk factors for aspiration, *N* = 1718		
Witnessed aspiration	332 (89.0)	41 (11.0)
Chronic impaired conscious level	67 (82.7)	14 (17.3)
Chronic neurologic disorders	411 (90.5)	43 (9.5)
Foreign bodies interfering with swallowing	19 (90.5)	2 (9.5)
Bed-ridden state	125 (84.5)	23 (15.5)
Body mass index, *N* = 1331		
< 18.5	333 (86.7)	51 (13.3)
18.5–24.9	724 (94.5)	42 (5.5)
≥ 25	175 (95.6)	8 (4.4)
CURB score, *N* = 1361		
0	477 (96.7)	16 (3.3)
1	464 (92.6)	37 (7.4)
2	219 (85.5)	37 (14.5)
3	80 (80.0)	20 (20.0)
4	5 (45.4)	6 (54.6)

*N, n* number of observations, *HCAP* healthcare-associated pneumonia, *CURB* Confusion, blood Urea nitrogen > 7 mmol/L, Respiratory rate > 30 per minute, and Blood pressure < 90 mmHg in systole or ≤ 60 mmHg in diastole

^a^patients who died within 30-day after the hospital visit

### Risk factors for 30-day mortality

In the univariate analyses, eleven underlying health conditions, including age ≥ 65 years, recent hospitalisation, nursing home residency, home care, male gender, neoplastic disease, dementia, witnessed aspiration, chronic impaired conscious level, bed-ridden state, and BMI < 18.5, were significant besides CURB score ≥ 1. In the multivariate analysis, six underlying health conditions were significant, independent of CURB score. These are home care (adjusted odds ratio (AOR), 5.84; 95% confidence interval (95% CI), 2.28–14.99), recent hospitalisation (2.21; 1.36–3.60), age ≥ 85 years (2.15; 1.08–4.28), low BMI (1.99, 1.25–3.16), neoplastic disease (1.82; 1.17–2.85), and male gender (1.78; 1.16–2.75). (Table 2).

**Table 2:** Risk factors for 30-day mortality

	30-day mortality			
	univariate analysis		multivariate analysis^b^	
	OR	95% CI	AOR	95% CI
Underlying health conditions				
Age group (years old)				
≤ 64	(1.00)		(1.00)	
65–74	2.46	1.27–4.74	1.61	0.80–3.26
75–84	2.49	1.37–4.53	1.37	0.70–2.69
≥ 85	3.98	2.21–7.16	2.15	1.08–4.28
HCAP criteria				
Hospitalization ≥2 days within 90 days	2.96	2.03–4.31	2.21	1.36–3.60
Nursing home resident	2.16	1.44–3.24	1.53	0.92–2.56
Chronic dialysis within 30 days	2.48	0.92–6.66		
Home care ^a^	7.00	2.69–18.23	5.84	2.28–14.99
Male gender	1.84	1.26–2.69	1.78	1.16–2.75
Comorbid illnesses				
Congestive heart failure	1.53	0.97–2.40		
Liver disease	1.75	0.93–3.29	1.77	0.87–3.61
Renal disease	1.54	0.92–2.61		
Neoplastic disease	2.18	1.49–3.18	1.82	1.17–2.85
Chronic lung disease	1.28	0.86–1.90		
Diabetes mellitus	1.22	0.80–1.86		
Dementia	1.69	1.09–2.63		
Risk factors for aspiration				
Witnessed aspiration	1.72	1.16–2.55		
Chronic impaired conscious level	2.71	1.28–4.98	1.75	0.81–3.76
Chronic neurologic disorders	0.80	0.37–1.76		
Foreign bodies interfering with swallowing	1.28	0.29–5.56	0.43	0.10–1.87
Bed-ridden state	2.49	1.53–4.06		
Body mass index				
< 18.5	2.63	1.72–4.04	1.99	1.25–3.16
18.5–24.9	(1.00)		(1.00)	
≥ 25	0.79	0.36–1.70	1.10	0.77–3.99
CURB score				
0	(1.00)		(1.00)	
1	2.38	1.30–4.33	1.87	1.01–3.46
2	5.04	2.74–9.25	3.65	1.95–6.82
3	7.45	3.71–14.99	4.90	2.22–10.81
4	35.78	9.88–129.59	29.14	6.07–139.74

*OR* odds ratio, *CI* confidence interval, *AOR* adjusted odds ratio, *HCAP* healthcare-associated pneumonia, *CURB* **C**onfusion, blood **U**rea nitrogen > 7 mmol/L, **R**espiratory rate > 30 per minute, and **B**lood pressure < 90 mmHg in systole or ≤ 60 mmHg in diastole

^a^Home care: home infusion therapy or wound care

^b^Variables after the variables with *p* > 0.2 had been removed by the backward stepwise method

The sensitivity analysis using bootstrap method showed almost the identical result to the derived model except that age ≥ 85 years and recent hospitalisation were not statistically significant. (Additional file 1: Table S1).

### Mortality prediction based on underlying health conditions, CURB65 and PSI

**Fig. 2:**
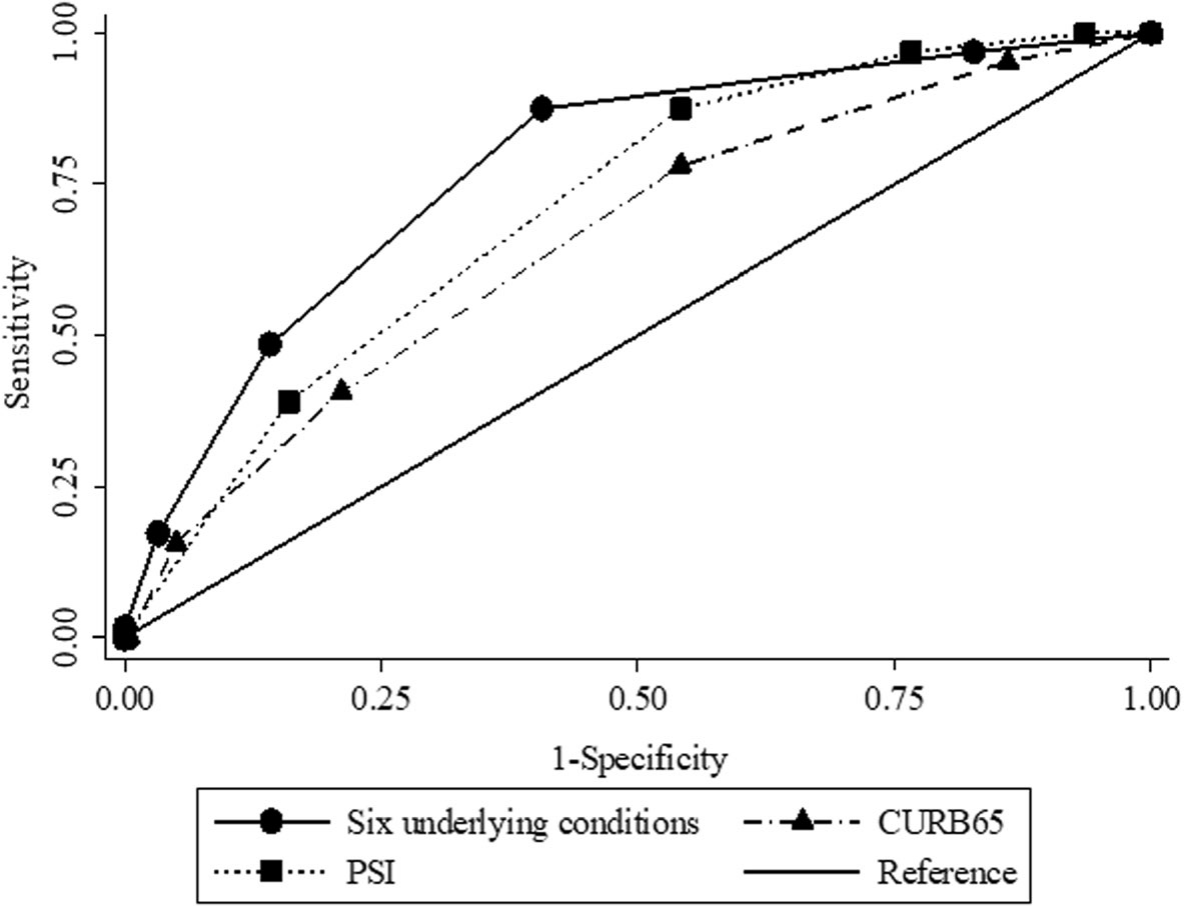
Mortality predictive index based on six underlying health factors had a higher AUROC curve compared with CURB65 and PSI. The AUROC curve of six underlying health factors was significantly higher than that of CURB65 (*p* = 0.02) and was marginally higher than that of PSI (*p* = 0.05). *CURB65* age ≥ **65** years, **C**onfusion, blood **U**rea nitrogen ≥7 mmol/L, **R**espiratory rate > 30 per minute, and **B**lood pressure < 90 mmHg in systole or ≤ 60 mmHg in diastole, *PSI* Pneumonia Severity Index, *AUROC* area under the receiver operating characteristic, *CI* confidence interval. Six underlying health conditions: age ≥ 85 years, hospitalization ≥2 days in the preceding 90 days, home care (wound care or infusion therapy at home), male gender, neoplastic disease, body mass index < 18.5

The number of patients without missing valuables of CURB65 and PSI was 836. Comparative analysis was conducted in these complete cases. The AUROC for the prediction index using six significant underlying health conditions alone was 0.78 (95% CI 0.72–0.83). It was significantly higher than that of CURB65 (AUROC 0.66, 95% CI 0.59–0.73; *p* = 0.02) and marginally higher than that of PSI (AUROC 0.71, 95% CI 0.66–0.77; *p* = 0.05) (Fig. 2). The AUROC of the total score consisting of six underlying health conditions and CURB score (range 0–10) was 0.79 (95% CI 0.74–0.85) and did not significantly improve mortality predictions compared to the index using the underlying health conditions alone (*p* = 0.3). (Additional file 2: FigureS1).

**Fig. 3:**
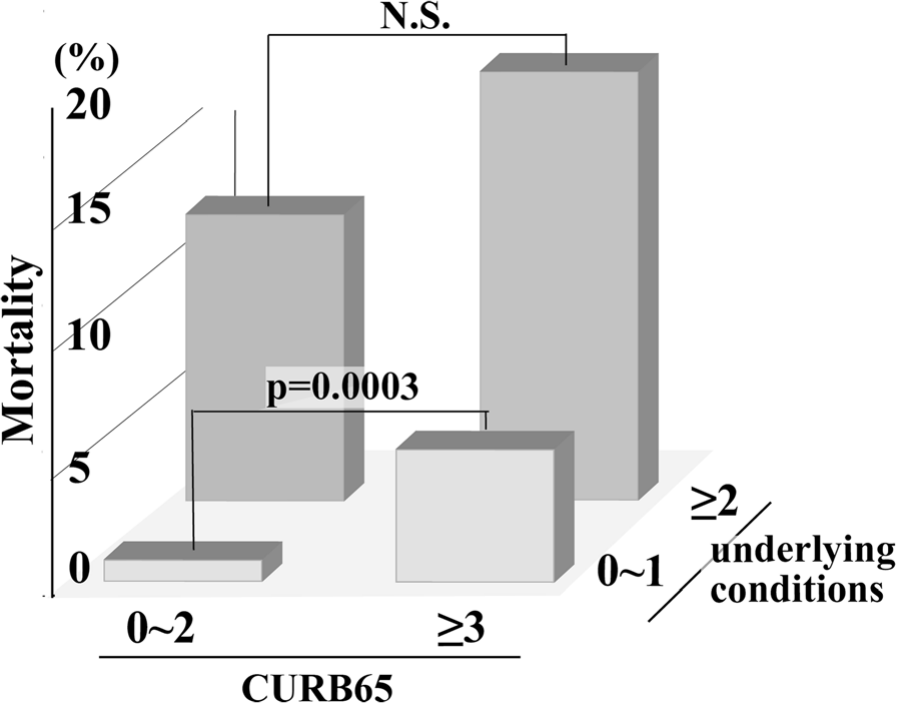
Mortality of mild and severe pneumonia according to the number of six underlying health conditions. The difference of morality between mild and severe pneumonia was not significant in patients with multiple underlying health conditions whereas mortality of severe pneumonia was significantly higher in patients with single or no underlying condition. *CURB65* age ≥ **65** years, **C**onfusion, blood **U**rea nitrogen ≥7 mmol/L, **R**espiratory rate > 30 per minute, and **B**lood pressure < 90 mmHg in systole or ≤ 60 mmHg in diastole, *N.S.* not significant

Patients were further stratified according to the number of underlying health conditions (0~ 1 vs ≥2) and the severity of pneumonia (mild vs severe pneumonia as defined by CURB65 < 3 vs ≥3, respectively). Among patients with 0~ 1 underlying health condition, 4 out of 425 (0.9%) patients with mild pneumonia and 6 out of 105 (5.7%) patients with severe pneumonia died; the mortality rate was discretely associated with the severity of pneumonia (risk ratio 7.24, 95% CI 3.08–25.13, *p* = 0.0003). On the other hand, among patients with ≥2 underlying health conditions, a high proportion of patients died regardless the severity of pneumonia: 33 out of 269 (12.3%) patients with mild pneumonia and 23 out of 125 (18.4%) patients with severe pneumonia died. In this group, the mortality rate was not significantly associated with the severity of pneumonia (risk ratio 1.53, 95% CI 0.94–2.50, *p* = 0.1). (Fig. 3). Because mortality risk may be confounded by do-not-resuscitate (DNR) orders [25], we excluded 62 patients who died without ventilator or vasopressor use, assuming that their deaths were affected by DNR orders. Removing those patients and analysing only the 78 non-survivors who were aggressively treated, did not change the results; the mortality of severe pneumonia was significantly higher than mild pneumonia (risk ratio 6.56, 95% CI 1.49–28.8, *p* = 0.004) in patients with 0~ 1 underlying health condition, whereas it was not significant in patients with ≥2 underlying health conditions (risk ratio 1.75, 95% CI 0.80–3.81, *p* = 0.2).

## Discussion

Analysing study population consisting predominantly of elderly patients, we identified six mortality-associated underlying health conditions: home infusion or wound care, recent hospitalisation, age ≥ 85 years, low BMI, neoplastic disease, and male gender, which are independent of a pneumonia severity score, such as CURB. We found those conditions influenced mortality independent of CURB65. CURB65 score better predicted mortality only when the patient had one or none of six conditions. In a systematic review, the AUROC of the CURB65 score was substantially lower and of the PSI score was also slightly lower in our study compared to those in most external validations (CURB65; about 0.8, PSI; slightly higher than 0.8) [26]. Chen et al. reported the underperformance of CURB65 and PSI in elderly patients [27]. In their study, excluding the age factor from CURB65 and PSI resulted in the increase in the AUROC curves, indicating that the age variable was inappropriately weighted and its cut-off value was also inappropriate to define the disease severity. In our study, approximately 75% of the study population was 65 years or older. This high proportion of elderly patients in our study could explain the reason of the underperformance of widely used severity scores. The risk difference on an absolute scale was similar in two groups: 2 or more underlying health conditions; 6.1%, 1or 0 underlying health condition; 4.8%. This may suggest that the CURB65 score may still be informative in patients with multiple underlying health conditions. However, as long as the mortality in patients with 2 or more underlying health conditions was extremely high even in patients with mild pneumonia (12.3%), we think that the high number of underlying health conditions should be identified along with severity assessment. Therefore, our results indicate that evaluating these underlying health conditions, along with pneumonia severity, will significantly improve mortality prediction in a society with an ageing population. If the number of underlying health conditions is one or less, CURB65 predicts mortality well and appropriate treatment is expected to improve survival outcomes; thus clinical management decisions should be guided accordingly. In our further analysis, the AUROC curve of CURB65 was 0.60 (95% CI: 0.52–0.68) in patients with two or more underlying health conditions, whereas it was 0.74 (95%CI: 0.58–0.90) in patients with 1 or 0 underlying health conditions (*p* = 0.12). Therefore, if the number of underlying health conditions is two or more, mortality is already high regardless of disease severity and CURB65 does not well predict the mortality of this patient group although the difference was not significant compared to that of patients with 1 or 0 underlying health condition.

Each of six conditions was also investigated in previous studies. Direct association between age or gender and mortality remains controversial [20, 21, 28]. However, age and gender are unchangeable values and whether they are independently associated with or confounded by the related conditions does not matter for prediction purposes. Our study population contained a substantial number of elderly patients and the result should be more appropriate to pneumonia patients in an ageing society. Most studies agree that HCAP itself is associated with mortality, though the reason for this association remains not well known [10, 29]. HCAP was originally proposed as a category of pneumonia associated with drug-resistant bacteria and higher mortality, but many studies argue against this association [30]. Thus in the present study, HCAP was not treated as a single category. Instead, each criterion was analysed as an individual condition. Similarly, the association between mortality and gender is also under debate. Presence of neoplastic disease is associated with both short-term and long-term mortality from pneumonia [31, 32]. PSI also includes neoplastic disease as a parameter, offering a high score of 30 points [11]. Low BMI increased mortality, whereas obesity was associated with better outcome; this so-called obesity paradox is recognised in pneumonia [22]. We understand that BMI is theoretically a modifiable parameter; however, in most clinical situations, it is difficult to improve BMI, especially for elderly people, which may reflect the presence of sarcopenia.

There was a marginally significant difference in the AUROC curve between the predictive index using six underlying health conditions and PSI. PSI was a better predictor of mortality than CURB65. This is probably because PSI contains not only disease parameters but also some underlying health conditions, such as nursing home residency and comorbid illnesses (neoplastic disease, congestive heart failure, cerebrovascular disease, renal disease, and liver disease). In addition, the PSI age variable is linearly scored and weighted according to the gender (10 points is subtracted from the age score of females). The inclusion of underlying health conditions might make PSI more suitable for morality prediction among pneumonia patients with increased age and multiple underlying health conditions.

Our study has some limitations. First, we did not perform the external validation using different cohort of pneumonia patients. This can cause the bias of a better performance of the derived model compared to the externally derived CURB and PSI scores. The validation study using a different cohort is necessary to generalise our conclusion. Second, although we showed an identical result from the sensitivity analysis, we did not validate our model internally using bootstrapped samples because we used a simple scoring method, 1 or 0 score in each variable, to develop the model. Therefore, overestimation of ORs and AUROC curves could not be assessed [33]. However, our initial concept was the model development in which primary care physicians can calculate it easily in a busy situation who are more likely to manage elderly patients with multiple underlying conditions. We kept our clinical concept for practical use of the model.

Third, we did not obtain detailed information about each underlying condition. In particular, neoplastic diseases should have been categorised according to their stages. Fourth, the mortality prediction model based on underlying health conditions was not validated on a different cohort of patients. Further studies using different cohorts of patients are necessary to validate our results. Last, we did not actively collect information about DNR orders in this study. However, we found a similar significance of the mortality risk ratio, even after excluding non-survivors without ventilator or vasopressor use, which we evaluated as a proxy for a DNR order. Long-term outcomes are also important to assess in patients with pneumonia in society with an ageing population.

## Conclusions

We have identified six underlying health conditions independently associated with 30-day mortality. The mortality was high in patients with multiple underlying health conditions, in which mortality prediction only by CURB65 score might not be accurate. We believe that co-evaluating underlying health conditions with disease severity has a significant benefit for pneumonia care in a society with an ageing population.

## Additional files

Additional file 1: **Table S1.** Sensitivity analysis using a bootstrapped dataset. The sensitivity analysis using bootstrap method showed almost the identical result to the derived model except that age ≥ 85 years and recent hospitalisation were not statistically significant. *AOR* adjusted odds ratio, *CI* confidence interval, *HCAP* healthcare-associated pneumonia, *CURB* **C**onfusion, blood **U**rea nitrogen > 7 mmol/L, **R**espiratory rate > 30 per minute, and **B**lood pressure < 90 mmHg in systole or ≤ 60 mmHg in diastole. ^a^ Home care: home infusion therapy or wound care. (DOCX 16 kb)

Additional file 2: **Figure S1.** The AUROC of the total score consisting of six underlying health conditions and CURB score. The AUROC of the total score consisting of six underlying health conditions and CURB score (range 0–10) was 0.79 (95% CI 0.74–0.85) and did not significantly improve mortality predictions compared to the index using the underlying health conditions alone (*p* = 0.3). (TIF 65 kb)

## Abbreviations

AUROC: Areas under the receiver operating characteristic curves; CI: confidence interval
BMI: Body mass index
BUN: Blood urea nitrogen
CAP: Community-acquired pneumonia
CT: Computed tomography
CURB: Confusion, elevated urea nitrogen, tachypnoea, and hypotension
CURB65: Confusion, elevated blood urea nitrogen, tachypnoea, hypotension, and age ≥ 65
CXR: Chest X-ray
DNR: Do-not-resuscitate
HCAP: Healthcare-associated pneumonia
IRB: Institutional Review Board
OR: Odds ratios
PSI: Pneumonia Severity Index
