# Supplementary material for: Six underlying health conditions strongly influence mortality based on pneumonia severity in an ageing population of Japan: a prospective cohort study
Source: BMC Pulm Med. 2018 May 23;18:88. doi: 10.1186/s12890-018-0648-y (PMC5967104; doi:10.1186/s12890-018-0648-y)
Supplement: Supplementary file 1 — Table S1. Sensitivity analysis using a bootstrapped dataset. The sensitivity analysis using bootstrap method showed almost the identical result to the derived model except that age ≥ 85 years and recent hospitalisation were not statistically significant. AOR adjusted odds ratio, CI confidence interval, HCAP healthcare-associated pneumonia, CURB Confusion, blood Urea nitrogen > 7 mmol/L, Respiratory rate > 30 per minute, and Blood pressure < 90 mmHg in systole or ≤ 60 mmHg in diastole. a Home care: home infusion therapy or wound care. (DOCX 16 kb) [file 12890_2018_648_MOESM1_ESM.docx]

**Supplementary table** Sensitivity analysis using a bootstrapped dataset

| Clinical factors | multivariate analysis | |
| --- | --- | --- |
|  | AOR | 95% CI |
| **Underlying health conditions** |  |  |
| Age group (years old) |  |  |
| ≤ 64 | (1.00) |  |
| 65 - 74 | 1.57 | 0.76 - 3.28 |
| 75 - 84 | 1.40 | 0.68 - 2.79 |
| ≥ 85 | 2.01 | 0.94 - 4.29 |
| HCAP factors |  |  |
| Hospitalisation ≥ 2 days in the preceding 90 days, | 1.29 | 0.77 - 2.17 |
| Nursing home resident | 1.53 | 0.92 - 2.56 |
| Chronic dialysis within 30 days | 1.71 | 0.35 - 8.39 |
| Home care^a^ | 5.69 | 1.46 - 22.27 |
| Male gender | 1.77 | 1.18 - 2.64 |
| Comobidities |  |  |
| Congestive heart failure | 1.02 | 0.61 - 1.71 |
| Liver disease | 1.80 | 0.80 - 4.06 |
| Renal disease | 1.11 | 0.51 - 2.40 |
| Neoplastic disease | 1.94 | 1.17 - 3.22 |
| Chronic lung disease | 1.21 | 0.76 - 1.91 |
| Diabetes mellitus | 1.10 | 0.70 - 1.75 |
| Dementia | 1.17 | 0.65 - 2.11 |
| Risk factors for aspiration-associated pneumonia |  |  |
| Witnessed aspiration | 0.97 | 0.54 - 1.74 |
| Chronic impaired conscious level | 1.70 | 0.63 - 4.58 |
| Chronic neurologic disorders | 0.48 | 0.10 - 2.35 |
| Foreign bodies interfering with swallowing | 1.60 | 0.78 - 3.26 |
| Bed-ridden state | 0.89 | 0.54 - 1.47 |
| Body mass index, N = 1331 |  |  |
| < 18.5 | 2.11 | 1.22 - 3.65 |
| 18.5 - 24.9 | (1.00) |  |
| ≥25 | 1.09 | 0.44 - 2.68 |
|  |  |  |
| **CURB score** |  |  |
| 0 | (1.00) |  |
| 1 | 1.82 | 0.99 - 3.38 |
| 2 | 3.47 | 1.82 - 6.60 |
| 3 | 5.13 | 2.09 - 12.59 |
| 4 | 23.18 | 3.34 - 160.74 |

*OR* odds ratio, *CI* confidence interval, *AOR* adjusted odds ratio, *HCAP* healthcare-associated pneumonia, *CURB* **C**onfusion, blood **U**rea nitrogen >7 mmol/L, **R**espiratory rate >30 per minute, and **B**lood pressure <90 mm Hg in systole or ≤60 mm Hg in diastole

^a^ Home care: home infusion therapy or wound care
