# Supplementary figures and images for: Six underlying health conditions strongly influence mortality based on pneumonia severity in an ageing population of Japan: a prospective cohort study
Source: BMC Pulm Med. 2018 May 23;18:88. doi: 10.1186/s12890-018-0648-y (PMC5967104; doi:10.1186/s12890-018-0648-y)

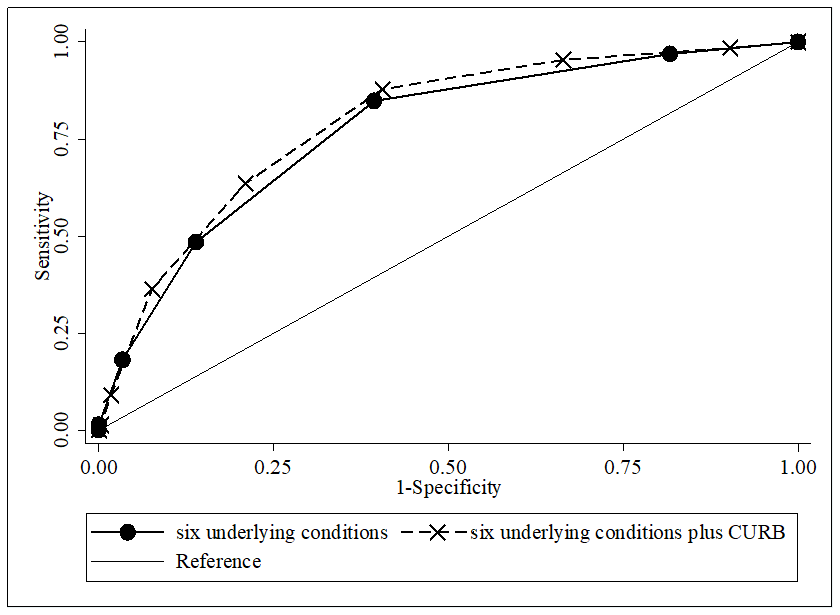

Supplement: Supplementary file 2 — Figure S1. The AUROC of the total score consisting of six underlying health conditions and CURB score. The AUROC of the total score consisting of six underlying health conditions and CURB score (range 0–10) was 0.79 (95% CI 0.74–0.85) and did not significantly improve mortality predictions compared to the index using the underlying health conditions alone (p = 0.3). (TIF 65 kb) [file 12890_2018_648_MOESM2_ESM.tif]
